# Pedicled hypothenar fat pad flap in recalcitrant carpal tunnel syndrome

**DOI:** 10.1186/1753-6561-9-S3-A73

**Published:** 2015-05-19

**Authors:** Christophe Mathoulin

**Affiliations:** 1 Institut de la Main, 75016, Paris, France

## Introduction

The incidence of failure in open carpal canal tunnel decompression is underestimated. Recurrence is often the result of scarring of the median nerve. Conservative treatment or careful neurolysis are usually insufficient.

## Methods

The hypothenar fat pad flap interposes adipose tissue from the hypothenar eminence and could offer a solution for patients with recalcitrant carpal tunnel syndrome. We reviewed the results of 56 patients with recalcitrant carpal tunnel syndrome using this procedure and analysed their subjective and objective results, pitfalls and complications.

## Results

For 51 patients (91%), the pain disappeared completely. Two-point discrimination improved from an expanded range to normal in 50 of the 56 patients (89%). Quick DASH (Disabilities of the Arm, Shoulder, and Hand) scores improved significantly.

## Discussion

The hypothenar fat pad flap is a technically simple procedure that prevents median nerve adherence producing excellent results and should be included in the repertoire of a surgeon performing recalcitrant carpal tunnel surgery.

